# Coupling effects of phosphorus fertilization source and rate on growth and ion accumulation of common bean under salinity stress

**DOI:** 10.7717/peerj.11463

**Published:** 2021-06-04

**Authors:** Heba I. Mohamed, Adel A. El-Sayed, Mostafa M. Rady, Gianluca Caruso, Agnieszka Sekara, Magdi T. Abdelhamid

**Affiliations:** 1 Biological and Geological Sciences Department, Faculty of Education, Ain Shams University, Cairo, Egypt; 2 Fertilization Technology Department, National Research Centre, Cairo, Egypt; 3 Botany Department, Faculty of Agriculture, Fayoum University, Fayoum, Egypt; 4 Department of Agricultural Sciences, University of Naples Federico II, Naples, Italy; 5 Department of Horticulture, Faculty of Biotechnology and Horticulture, University of Agriculture in Krakow, Krakow, Poland; 6 Botany Department, National Research Centre, Cairo, Egypt

**Keywords:** Mineral content, *Phaseolus vulgaris* L., Salt stress, Superphosphate, Urea phosphate

## Abstract

Many agricultural regions in arid and semiarid climate zone need to deal with increased soil salinity. Legumes are classified as salt-sensitive crops. A field experiment was performed to examine the application of phosphorus (P) fertilizer source and rate on growth, chlorophylls and carotenoid content, DNA and RNA content and ion accumulation in common bean (*Phaseolus vulgaris* L.) cultivated under salinity stress. An experimental design was split-plot with three replicates. The main plots included two P sources, namely single superphosphate (SP) and urea phosphate (UP). The sub-plots covered four P rates, i.e., 0.0, 17.5, 35.0, and 52.5 kg P ha^–1^. All applied P fertilization rates, in both forms, increased plant height, leaf area, dry weight of shoots and roots per plant, and total dry weight (TDW) in t ha^−1^. The highest accumulation of N, P, K^+^, Mg^2+^, Mn^2+^, Zn^2+^, and Cu^2+^ was determined in the shoot and root of common bean, while 35 kg of P per ha^−1^ was used compared to the other levels of P fertilizer. The highest P rate (52.5 kg ha^−1^) resulted in a significant reduction in Na^+^ in shoot and root of common bean. The response curve of TDW (t ha^–1^) to different rates of P (kg ha^–1^) proved that the quadratic model fit better than the linear model for both P sources. Under SP, the expected TDW was 1.675 t ha^–1^ if P was applied at 51.5 kg ha^–1^, while under UP, the maximum expected TDW was 1.875 t ha^–1^ if P was supplied at 42.5 kg ha^–1^. In conclusion, the 35.0 kg P ha^–1^ could be considered the best effective P level imposed. The application of P fertilizer as urea phosphate is generally more effective than single superphosphate in enhancing plant growth and alleviating common bean plants against salinity stress.

## Introduction

From an agricultural point of view, the beginning of the 21^st^ century is characterized by global warming, rainfall deficit, environmental deterioration, and increased soil and water salinization. Sustainable agriculture has to tackle two major issues: the rising human population and the decrease the area of land available for strategic crops’ cultivation (Shahbaz & Ashraf, 2013). It was estimated that 20% of total crop production worldwide and 33% of irrigated agricultural land in Egypt is under pressure of high salinity (Akladious & Mohamed, 2018; Sofy, Elhawat & Alshaal, 2020a). Besides, the salinized areas have been rising at a rate of 10% annually as a result of low rainfall, high surface evaporation, soil erosion, saline water irrigation, and extensive cultural practices. It is forecasted that by the year 2050 more than half of the arable area would be salinized (Jamil et al., 2011; Latif & Mohamed, 2016; Mohamed, Akladious & El-Beltagi, 2018).

The salinity stress in crops arise from the interactions between morphological, physiological, and biochemical processes affecting primarily germination stage and plant development and productivity through disturbed water and nutrient absorption, and enzyme activity (Abdelhamid et al., 2013; El-Mashad & Mohamed, 2012; Mohamed & Gomaa, 2012), DNA, RNA, and protein synthesis, and proper course of mitosis in cells (Javid et al., 2011). It was observed that as salinity (NaCl, CaCl_2_) increased, phosphate concentrations decreased in field-grown agricultural crops (Baker et al., 2013). Besides, Curtin, Steppuhn & Selles (1993) reported that increased Na^+^ and Ca^2+^ concentrations in the soil solution can contribute to raised ionic strength in the soil that can improve P retention. The other elements and ions, including sodium (Na^+^), chlorine (Cl^‒^), and boron (B) have particularly toxic effects on plants (Moustafa-Farag et al., 2020). The accumulation of sodium causes disturbances of cell membranes permeability, followed by osmotic stress and cell death (Sadak & Abdelhamid, 2015).

In salinity conditions, ion toxicity results from the substitution of potassium (K^+^) by Na^+^ in biochemical reactions, and conformation disturbances in protein structure caused by Na^+^ and Cl^‒^. Notably, K^+^ serves as a cofactor for many enzymes, and Na^+^ ions are not able to take over as K^+^. High concentration of K^+^ is necessary to bind tRNA to ribosome in protein synthesis process (Rozov et al., 2019). Na^+^ toxicity, followed by osmotic stress, contribute to metabolic dysfunction, resulting in oxidative stress (Orabi & Abdelhamid, 2016). During the reproductive process, the salinity stress is the most destructive, concerning plant growth parameters.

Phosphorus (P) is mentioned among the most essential plant growth nutrients, since it is a crucial element of biological compounds, such as nucleic acids, nucleotides, phospholipids, and phospho-proteins. Phosphate compounds function inside plants as the “carbon currency” (Ahmed et al., 2018). Additional P application mitigates the reduction of plant growth caused by salinity (Wagdi et al., 2013). Under salt stress conditions, higher P dose caused a reduction in the Na^+^ content in wheat (Wagdi et al., 2013), common bean (Bargaz et al., 2016), and radish (Oliveira et al., 2010). Therefore, the interaction between salinity and P nutrition is multifaceted, and it depends on species or cultivar, stage of growth, salt composition and concentration, and the content of P in soil (Fageria, Gheyi & Moreira, 2011).

While several studies examined the impact of salinity or P deficiency on plant growth and nutrient uptake as independent growth-limiting factors, the knowledge on their combined action is still insufficient (Bargaz et al., 2016). Munns & James (2003) determined that durum wheat absorbed the amount of P, exciding toxic level, in saline solution. Bargaz et al. (2016) investigated the grown response of common bean to three salinity levels (1.56, 4.78, and 8.83 dS m^–1^) and four P rates (0, 30, 60, and 90 kg ha^–1^), and reported improved salt tolerance through adequate P fertilization. *P. vulgaris* tolerance against salinity appears to be P-rate dependent. Abbas et al. (2018) found that low P (10 M) was more harmful to root and shoot growth of wheat than 100 mM NaCl, considering their discrepancies in salinity tolerance.

Common bean (*Phaseolus vulgaris* L.) is a grain food legume of economic importance in Egypt and many other countries (Broughton et al., 2003). Besides, *P. vulgaris* contributes about 50% of the world’s grain legumes consumed, supplying high-quality protein in the human diet (Graham & Vance, 2003). Unfortunately, the common bean is a salt-sensitive species, reacting in significant growth reduction at soil salinity less than 2 dS m^–1^ (Lauchli, 1984).

The hypothesis verified in this study was that with application of P in different P forms and various P rates, common bean plants show increased growth parameters under P deficiency and salinity stress. To verify this issue, a field experiment was performed, covering two forms of P: single superphosphate and urea phosphate, and four P fertilization rates: 0.0, 17.5, 35.0, and 52.5 kg P ha^–1^. Growth parameters, chlorophyll content, nucleic acid content and mineral nutrients in common bean (*Phaseolus vulgaris* L.), cultivated in conditions of P deficiency and saline stress were examined.

## Materials & Methods

### Experimental procedures

A field experiment was performed in moderate saline soil at a private farm, El Qanater El Khayreya, Qalyubia Governorate, Egypt (31°09′07″E 30°11′35″N) during the 2019 season. daily temperatures ranged from 16.8 to 36.4 °C with an average of 25.4±4.8 °C, while night-time temperatures 9.2 ± 3.8 °C, with a minimum and a maximum of 2.2 and 18.2 °C. The average daily relative humidity was 68.7 ± 10.3% and ranged between 36.6% and 83.7%.

The common bean seeds (*Phaseolus vulgaris* L. cv. Nebraska) were selected, uniform and representative concerning size and color. The seeds were washed with distilled water, sterilized with 1.0% sodium hypochlorite solution for 2 min, washed with distilled water, and dried for 1 h at room temperature. *Rhizobial* inoculants have been used as peat slurry that contains 10^7^ *Rhizobium* per gram. The recommended seed rate for the common bean of 95 kg ha^–1^ was used. Common bean seeds were sown in the field on the 10^th^ March 2019. Before the first irrigation, plants were thinned and one plant per hill was left. Before the cultivation, ammonium sulfate and potassium sulfate, at rates of 50 kg N ha^–1^ and 125 kg K ha^–1^ were supplemented according to the recommendations for the species. Before sowing, the physical and chemical soil characteristics were analysed with the methods described by Black et al. (1965) and Jackson (1973), and the results were presented in Table 1. Soil paste extract was used to determine electrical conductivity (EC). EC_e_ was 5.21 dS m^–1^ that defines the soil as moderately saline (Hazelton & Murphy, 2016). Traditionally, saline soils are classified as having an EC_e_ value greater than 4 dS m^–1^. Salinity is an EC_e_-based rating system for soil. When the EC_e_ value varied between 4 and 8 dS m^–1^, the soil is classified as moderately saline. As a result, salt has an effect on the yields of many crops (Hazelton & Murphy, 2016). Extractable soil phosphorus (P) content was 7.02 mg kg^–1^ soil, which classed the soils as being low in P (Holford & Cullis, 1985).

**Table 1:** Physical and chemical properties of the experimental soil before common bean sowing.

Parameter	Value
Physical:	
Clay (%)	55
Silt (%)	25
Sand (%)	20
Soil texture	Clay loam
Field capacity (%)	32.9
Chemical:	
pH	8.35
EC_e_ (dS m^–1^)	5.21
Organic matter (%)	1.67
CaCO_3_ (%)	7.88
Available nutrients (mg kg^–1^ soil)	
N (NO_3_-N)	13.1
N (NH_3_-N)	18.2
P	7.02
K	181.0
Ca	750.0
Mg	105.0
S	8.0
Fe	5.9
Mn	1.07
Zn	0.78
Cu	0.57

The experiment was conducted using a split-plot arrangement in a randomized complete block design with three replicates. P source was the first factor, assigned to the main plots, the rate of P was a second factor, allocated to the sub-plots (split-plots). P sources were as follows: (1) single superphosphate (SP), and (2) urea phosphate (UP). P rates were following: 0.0, 17.5, 35.0, and 52.5 kg P ha^–1^. Each replicate included eight plots, i.e., two P sources × four P rates, eight treatments in total as it was shown in Tables 2–7. P fertilizer was added to the soil during its preparation for cultivation. Single superphosphate (SP) contained 15.5% phosphorus pentoxide P_2_O_5_, 19.5% calcium, 11.5% sulphur, and 2.0 pH, while urea phosphate (UP) contained 17.5% total nitrogen, 44.0% P_2_O_5_, 2.0 pH, and 10% solution. P fertilizer was applied to the soil before sowing. Experimental plots consisted of 5 rows, 4 m long and 0.75 m wide, with the hills which were spaced 4–5 cm apart. Plots were irrigated using the reference crop evapotranspiration (ET0) values. All other agricultural practices were performed according to the recommendations of the Ministry of Agriculture and Land Reclamation, Egypt for common bean and referred literature (Abdelhamid et al., 2013).

**Table 2:** Interactive coupling effects of phosphorus fertilization source and rate on total chlorophylls (TC) and carotenoids (Car) of common bean plants grown under salinity stress.

Treatments		Total chlorophylls	Carotenoids
P source	P rate	(mg g^–1^ FW)	
SP	0.0	0.453^†^^e^	0.313^e^
	17.5	0.467^e^	0.326^e^
	35.0	0.548^bc^	0.414^c^
	52.5	0.495^d^	0.357^d^
UP	0.0	0.542^c^	0.404^c^
	17.5	0.566^bc^	0.455^b^
	35.0	0.632^a^	0.513^a^
	52.5	0.571^b^	0.465^b^
Significance of F test:			
P source		**	**
P rate		**	**
P source × P rate		ns	**

**Note:**

† Mean values within the same column for each trait with the same lower case letter are not significantly different according to Tukey’s honestly significant difference (HSD) test at *P* ≤ 0.05. ** and *, significant at 0.01 and 0.05 levels respectively; ns, non-significant. Measurements were done at 56 days after sowing. SP, superphosphate; UP, urea phosphate; P source, phosphorus source; P rate, phosphorus application rate 0.0, 17.5, 35.0, and 52.5 kg ha^–1^.

**Table 3:** Interactive coupling effect of phosphorus fertilization source and rate on the nucleic acids (DNA and RNA) of common bean plants grown under salinity stress.

Treatments		RNA	DNA
P source	P rate	(mg 100 g^–1^ DW)	
SP	0.0	805^†^^d^	364^e^
	17.5	941^cd^	441^d^
	35.0	1,125^ab^	518^b^
	52.5	1057^bc^	458^d^
UP	0.0	890^cd^	386^e^
	17.5	1,029^bc^	472^cd^
	35.0	1,264^a^	608^a^
	52.5	1,131^ab^	507^bc^
Significance of F test:			
P source		**	*
P rate		**	**
P source × P rate		ns	**

**Note:**

† Mean values within the same column for each trait with the same lower case letter are not significantly different according to Tukey’s honestly significant difference (HSD) test at *P* ≤ 0.05. ** and *, significant at 0.01 and 0.05 levels respectively; ns, non-significant. Measurements were done at 56 days after sowing. SP, superphosphate; UP, urea phosphate; P source, phosphorus source; P rate, phosphorus application rate 0.0, 17.5, 35.0, and 52.5 kg ha^–1^.

**Table 4:** Interactive coupling effect of phosphorus fertilization source and rate on macro-nutrients (N, P, K^+^, Ca^2+^, Mg^2+^) and Na^+^ of common bean plants grown under salinity stress.

Treatments		N	P	K^+^	Ca^2+^	Mg^2+^	Na^+^	N	P	K^+^	Ca^2+^	Mg^2+^	Na^+^
P source	P rate	Shoot (g kg^−1^)						Root (g kg^−1^)					
SP	0.0	27.2^†^ ^d^	1.90 ^e^	22.1 ^de^	18.8 ^g^	9.43 ^e^	0.59 ^a^	20.1 ^a^	1.03 ^e^	8.2 ^e^	0.40 ^b^	3.50 ^cd^	0.93 ^a^
	17.5	33.3 ^bc^	2.10 ^cde^	23.0 ^abc^	20.2 ^ef^	10.60 ^cd^	0.52 ^b^	18.4 ^b^	1.20 ^e^	9.6 ^cd^	0.47 ^ab^	3.87 ^abc^	0.87 ^b^
	35.5	35.0 ^ab^	2.40 ^abc^	23.3 ^abc^	21.6 ^cd^	11.23 ^bc^	0.46 ^c^	15.8 ^d^	1.50 ^cd^	11.7 ^a^	0.57 ^ab^	3.97 ^ab^	0.80 ^cd^
	52.5	32.6 ^c^	2.37 ^abc^	21.2 ^e^	23.6 ^b^	9.67 ^e^	0.42 ^d^	14.4 ^e^	1.87 ^ab^	10.1 ^c^	0.50 ^ab^	4.20 ^a^	0.71 ^e^
UP	0.0	29.1 ^d^	2.00 ^de^	22.8 ^bcd^	19.4 ^fg^	9.73 ^de^	0.58 ^a^	19.5 ^a^	1.10 ^e^	8.7 ^de^	0.47 ^ab^	3.27 ^d^	0.90 ^ab^
	17.5	34.4 ^abc^	2.33 ^bcd^	23.8 ^a^	21.0 ^de^	11.77 ^ab^	0.50 ^b^	17.0 ^c^	1.30 ^de^	10.5 ^bc^	0.47 ^ab^	3.73 ^bc^	0.82 ^c^
	35.0	35.7 ^a^	2.70 ^a^	23.6 ^ab^	22.4 ^c^	12.27 ^a^	0.44 ^c^	15.4 ^d^	1.70 ^bc^	11.4 ^ab^	0.63 ^a^	3.77 ^bc^	0.77 ^d^
	52.5	33.9 ^abc^	2.63 ^ab^	22.3 ^cd^	24.8 ^a^	10.73 ^c^	0.41 ^d^	13.8 ^e^	2.03 ^a^	8.8 ^de^	0.47 ^ab^	3.90 ^abc^	0.70 ^e^
Significance of F test:													
P form		**	*	**	*	*	**	*	**	ns	ns	ns	ns
P rate		**	**	**	**	**	**	**	**	**	**	**	**
P source × P rate		ns	ns	ns	ns	ns	ns	*	ns	**	ns	ns	ns

**Note:**

† Mean values within the same column for each trait with the same lower case letter are not significantly different according to Tukey’s honestly significant difference (HSD) test at *P* ≤ 0.05. ** and *, significant at 0.01 and 0.05 levels respectively; ns, non-significant. Measurements were done at 56 days after sowing. SP, superphosphate; UP, urea phosphate; P source, phosphorus source; P rate, phosphorus application rate 0.0, 17.5, 35.0, and 52.5 kg ha^–1^. N, nitrogen; P, phosphorus; K^+^, potassium; Ca^2+^, calcium; Mg^2+^, magnesium; Na^+^, sodium.

**Table 5:** Interactive coupling effect of phosphorus fertilization source and rate on micro-nutrients (Fe^3+^, Mn^2+^, Zn^2+^, and Cu^2+^) of common bean plants grown under salinity stress.

Treatments		Fe^3+^	Mn^2+^	Zn^2+^	Cu^2+^	Fe^3+^	Mn^2+^	Zn^2+^	Cu^2+^
P source	P rate	Shoot (mg kg^−1^)				Root (mg kg^−1^)			
SP	0.0	278^†^ ^d^	57 ^d^	58.7 ^d^	10.3 ^e^	2066 ^a^	84 ^a^	30.0 ^d^	16.0 ^a^
	17.5	570 ^c^	68 ^c^	61.8 ^cd^	11.3 ^cde^	1840 ^b^	73 ^b^	35.8 ^c^	14.2 ^ab^
	35.0	618 ^b^	89 ^b^	67.5 ^ab^	13.3 ^ab^	1433 ^c^	51 ^c^	37.3 ^bc^	13.0 ^bcd^
	52.5	731 ^a^	94 ^a^	63.7 ^bcd^	11.5 ^cde^	1247 ^e^	42 ^de^	38.3 ^ab^	12.2 ^cd^
UP	0.0	286 ^d^	62 ^d^	60.4 ^cd^	10.6 ^de^	2016 ^a^	81 ^a^	31.3 ^d^	15.3 ^a^
	17.5	583 ^c^	73 ^c^	63.7 ^bcd^	12.8 ^bc^	1806 ^b^	71 ^b^	36.8 ^bc^	13.3 ^bc^
	35.0	633 ^b^	92 ^ab^	69.3 ^a^	15.0 ^a^	1357 ^d^	46 ^d^	38.0 ^ab^	12.7 ^bcd^
	52.5	741 ^a^	96 ^a^	65.3 ^abc^	12.2 ^bcd^	1199 ^e^	39 ^e^	39.4 ^a^	11.3 ^d^
Significance of F test:									
P form		*	*	ns	ns	ns	ns	*	**
P rate		**	**	**	**	**	**	**	**
P form × P rate		ns	ns	ns	ns	ns	ns	ns	ns

**Note:**

† Mean values within the same column for each trait with the same lower case letter are not significantly different according to Tukey’s honestly significant difference (HSD) test at *P* ≤ 0.05. ** and *, significant at 0.01 and 0.05 levels respectively; ns, non-significant. Measurements were done at 56 days after sowing. SP, superphosphate; UP, urea phosphate; P source, phosphorus source; P rate, phosphorus application rate 0.0, 17.5, 35.0, and 52.5 kg P ha^–1^. Fe^3+^, iron; Mn^2+^, manganese; Zn^2+^, zinc; Cu^2+^, copper.

**Table 6:** Interactive coupling effect of phosphorus fertilization source and rate on K^+^:Na^+^ and Ca^2+^:Na^+^ ratios in shoots and roots of common bean plants grown under salinity stress.

Treatments		K^+^:Na^+^ ratio	Ca^2+^:Na^+^ ratio	K^+^:Na^+^ ratio	Ca^2+^:Na^+^ ratio
P source	P rate	Shoot		Root	
SP	0.0	37.6^†^ ^e^	32.1 ^g^	8.9 ^e^	0.43 ^c^
	17.5	44.3 ^d^	38.9 ^f^	11.0 ^d^	0.54 ^bc^
	35.0	50.2 ^b^	46.7 ^d^	14.6 ^a^	0.71 ^ab^
	52.5	50.9 ^b^	56.6 ^b^	14.2 ^ab^	0.71 ^ab^
UP	0.0	39.6 ^e^	33.7 ^g^	9.6 ^de^	0.52 ^bc^
	17.5	47.6 ^c^	41.9 ^e^	12.8 ^bc^	0.57 ^abc^
	35.5	53.3 ^a^	50.5 ^c^	14.9 ^a^	0.83 ^a^
	52.5	54.9 ^a^	60.9 ^a^	12.6 ^c^	0.67 ^abc^
Significance of F test:					
P source		*	*	ns	ns
P rate		**	**	**	**
P source × P rate		ns	ns	**	ns

**Note:**

† Mean values within the same column for each trait with the same lower case letter are not significantly different according to Tukey’s honestly significant difference (HSD) test at *P* ≤ 0.05. ** and *, significant at 0.01 and 0.05 levels respectively; ns, non-significant. Measurements were done at 56 days after sowing. SP, single superphosphate; UP, urea phosphate; P source, phosphorus source; P rate, phosphorus application rate 0.0, 17.5, 35.0, and 52.5 kg ha^–1^.

**Table 7:** Interactive coupling effect of phosphorus fertilization source and rate on growth and biomass yield of common bean plants grown under salinity stress.

Treatments	P rate	PH	LA	SDW	RDW	TDW	TDW
P source		(cm)	(dm^2^ plant^–1^)	(g plant^–1^)	(g plant^–1^)	(g plant^–1^)	(t ha^–1^)
SP	0.0	27.9^†^ e	2.05 h	2.83 g	0.28 d	3.11 g	0.934 g
	17.5	31.2 cde	2.88 f	3.46 f	0.39 c	3.86 f	1.157 f
	35.0	35.2 abc	4.19 c	5.24 c	0.52 b	5.76 c	1.727 c
	52.5	33.2 bcd	3.82 d	4.73 d	0.44 c	5.17 d	1.552 d
UP	0.0	29.6 de	2.59 g	3.14 fg	0.33 d	3.47 fg	1.041 fg
	17.5	33.0 bcd	3.41 e	4.12 e	0.43 c	4.56 e	1.367 e
	35.0	38.0 a	5.43 a	6.35 a	0.65 a	7.01 a	2.102 a
	52.5	36.7 ab	4.94 b	5.67 b	0.54 b	6.21 b	1.864 b
Significance of F test:							
P source		**	**	**	**	**	**
P rate		**	**	**	**	**	**
P source × P rate		ns	**	**	**	**	**

**Note:**

† Mean values within the same column for each trait with the same lower case letter are not significantly different according to Tukey’s honestly significant difference (HSD) test at *P* ≤ 0.05. ** and *, significant at 0.01 and 0.05 levels respectively; ns, non-significant. ** and *, significant at 0.01 and 0.05 levels respectively; ns, non-significant. Measurements were done at 56 days after sowing. SP, single superphosphate; UP, urea phosphate; P source, phosphorus source; P rate, phosphorus application rate 0.0, 17.5, 35.0, and 52.5 kg ha^–1^. PH, plant height; LA, leaf area; SDW, shoot dry weight; RDW, root dry weight, TDW, total dry weight.

### Measurements

At 56 day after sowing (DAS), the following characteristics were analyzed on the samples consisted of ten plants per treatment: leaf area per plant, root dry weight per plant, shoot dry weight per plant, and total dry weight (DW) per plant. Total dry weight yield (t ha^–1^) was determined by harvesting all plants from each experimental unit.

Chlorophylls and carotenoids were determined in the fresh leaves of 56 day-old plants. Pigments were extracted by homogenizing of 100 mg of fresh leaf tissue in pestle and mortar, in 80% acetone solution (10 mL) followed by centrifugation for 20 min at 3,000 *g*. The samples were storage at 4 °C for night, then optical density was measured at 480 nm for carotenoids, and 645 and 663 nm for chlorophyll a and b, respectively, following the method described by Arnon (1949).

DNA and RNA were extracted from leaves of common bean using a bench drill according to cetyltrimethylammonium bromide (CTAB) protocol (Doyle & Doyle, 1990) and (Ferreira et al., 2019) for DNA and RNA extractions, respectively. The grinded plant tissues were dissolved inside the plastic bags (20 × 10 × 0.01 cm of virgin, low-density polyethylene) at rotation of 2,500 rpm, until tissue is fully dissolved. The drill bit was cleaned with 70% alcohol for each sample change. The extraction buffer consisted of 2% CTAB, 100 mM, Tris-HCl, pH 8.0, 50 mM EDTA, pH 8.0; 1.4 M NaCl, 2% PVP-40; 1% Na sulfite, all reagents were add before use. Two mL of extraction buffer were added to plastic bags containing 300 mg of leaf tissue and macerated with Biorema drill (Agdia, Inc., Elkhart, IN, USA).

Dried roots and shoots of 56 day-old plants were used for determining the ion accumulation. The samples were dried at 70 °C, in an electric oven, till constant weight was accessed, and finally ground. Nutrients concentration was determined in the shoot and root of common bean plants. Macronutrients namely, nitrogen (N), P, K^+^, calcium (Ca^2+^), and magnesium (Mg^2+^), and micro-nutrients: iron (Fe^3+^), manganese (Mn^2+^), zinc (Zn^2+^), and copper (Cu^2+^), in addition to sodium (Na^+^) were determined. Total N was analyzed with the micro-Kjeldahl method. P was determined colorimetrically, after P extraction by sodium bicarbonate (Olsen, 1954), with stannous chloride-ammonium molybdate (King, 1951). K^+^ was measured with a flame photometer (ELE Flame Photometer, Leighton Buzzard, UK). Ca^2+^, Mg^2+^, Fe^3+^, Mn^2+^, Zn^2+^, Cu^2+^, and Na^+^ concentrations were determined by atomic absorption spectrophotometry (Chapman, 1965). N, P, K^+^, Ca^2+^, Mg^2+^, and Na^+^ were expressed as gram per kilogram (g kg^−1^), while Fe^3+^, Mn^2+^, Zn^2+^, and Cu^2+^ were expressed as milligram per kilogram (mg kg^−1^).

### Statistical analysis of the data

The analysis of variance for a split-plot design was used to statistical elaboration of the experimental data (Gomez & Gomez, 1984). Before the analysis, the data were tested for the homogeneity of error variances with the Levene test (Levene, 1961), and for normality distribution with Shapiro and Wilk method (Shapiro & Wilk, 1965). Statistically significant differences between means were determined at the 5% probability level (*P* ≤ 0.05) using Tukey’s HSD (honestly significant difference) test. Correlation coefficients *r* were calculated to determine the relationship between biomass dry weight yield and each of the mineral concentrations. The GenStat 17^th^ Edition (VSN International Ltd., Hemel Hempstead, UK) software was used for statistical analysis.

## Results

To examine the effects of P fertilization applied in the form of superphosphate and urea phosphate, and P fertilization rate on common bean grown under salt-affected soil, thirty four traits were measured and presented below.

### Photosynthetic pigments of common bean

The interactive effect of phosphorus fertilization source and rate on total chlorophylls (TC) and carotenoids (Car) of common bean plants grown under salinity stress were shown in Table 2. Besides, the significance of the F test of analysis of variance was shown in Table 2. Urea Phosphate (UP) significantly increased TC and Car compared to superphosphate (SP) by 17.7% and 30.0%, respectively. The main effects of P rates were highly significant (*P* ≤ 0.01) and affected TC and Car. Application of P in a dose of 35.0 kg P ha^–1^ compared to the control significantly increased TC and Car by 18.5% and 29.2%, respectively. TC increased gradually with increasing P rate and application of 35.0 kg P ha^–1^ in SP and UP source caused the highest values by 20.9% and 16.7%, respectively compared to the control. Also, Car increased progressively with increasing P rate, the application of 35.0 kg P ha^−1^ resulted in the greatest values in SP and UP source by 32.4% and 27.0%, respectively.

### DNA and RNA of common bean

Table 3 shows the interactive effect of phosphorus fertilization source and rate on the nucleic acids (DNA and RNA) of common bean plants grown under salinity stress. Moreover, the significance of the F test of analysis of variance was shown in Table 3. Urea phosphate significantly increased RNA and DNA compared to SP by 9.8% and 10.8%, respectively. The main effects of P rates were highly significant (*P* ≤ 0.01) and affected RNA and DNA. Application of P with 35.0 kg P ha^–1^ compared to control significantly increased RNA and DNA by 40.9% and 50.1%, respectively. RNA increased gradually with increasing P rate and application of 35.0 kg P ha^–1^ in SP and UP source caused the highest values by 39.8% and 42.0%, respectively compared to the control. Also, DNA increased gradually with increasing P rate and application of 35.0 kg P ha^–1^ resulted in greatest values in SP and UP source by 42.3% and 57.2%, respectively.

### Ion concentration of common bean

The interactive effect of different sources and rates of phosphorus fertilization on macro-nutrients (N, P, K^+^, Ca^2+^, Mg^2+^), and Na^+^ of common bean plants grown under salt-affected soil (5.21 dS m^–1^) were shown in Table 4. The significance of the *F* test of analysis of variance was shown also in Table 4. The main effects of P source were significant (*P* ≤ 0.05) in shoot N, shoot Ca^2+^, shoot Mg^2+^ and root N, highly significant (*P* ≤ 0.01) in shoot N, shoot K^+^, shoot Na^+^, and root P, while was non-significant in root K^+^, root Ca^2+^, root Mg^2+^, and root Na^+^. Application of UP increased all macro-nutrients in both shoot and roots, while reduced Na^+^ in shoot and root as well, compared to SP. The main effects of P rates were highly significant (*P* ≤ 0.01) and affected in all aforesaid traits as shown in Table 4. However, P reduced Na^+^ in shoots and roots and this reduction was in parallel with increasing P rate to the dose of 52.5 kg P ha^–1^. Under the interactive effect of P source and P rate, application of P increased N, P, K^+^, and Mg^2+^ of shoots till 35.0 kg P ha^–1^, whereas shoot Ca^2+^ increased gradually to the dose of 52.5 kg P ha^–1^. In roots, application of P enhanced N, K^+^, Ca^2+^ to the dose of 35.0 kg P ha^–1^, while 52.5 kg P ha^–1^ decreased these nutrients compared to 35.0 kg P ha^–1^. However, the P application increased P and Mg to 52.5 kg P ha^–1^. Application of P boosted P concentration in shoots by 26.3% and 42.1% under SP, and UP, respectively when P in a dose of 35.0 kg P ha^–1^ was applied. Moreover, P application resulted in a reduction in shoot Na^+^ by 27.6% and 29.3% in SP, and UP, respectively, while the reduction in roots Na^+^ was by 21.1% and 22.2% in SP, and UP, respectively. The response in Na^+^ concentration as P application reduced Na^+^ gradually with increasing P rate regardless of P source. The highest P rate resulted in the lowest Na^+^ concentration either in shoots or roots. The response due to P application may refer to P rate more than P source.

Table 5 shows the interactive effect of different sources and rates of phosphorus fertilization on micro-nutrients (Fe^3+^, Mn^2+^, Zn^2+^, Cu^2+^), in shoots and roots of common bean plants grown under salt-affected soil (5.21 dS m^–1^). The significance of the F test of analysis of variance was shown also in Table 5. The main effects of P source were significant (*P* ≤ 0.05) in shoot Fe^3+^, shoot Mn^2+^, root Zn^2+^, and highly significant (*P* ≤ 0.01) in root Cu^2+^. P application in UP source increased Fe^3+^, Mn^2+^, Zn^2+^, and Cu^2+^ in shoot and Zn^2+^ in root compared to SP source (Table 5). The main effects of P rates were highly significant (*P* ≤ 0.01) and affected in all micro-nutrients in shoots and roots as shown in Table 5. Application of P increased Fe^3+^, Mn^2+^, Zn^2+^, and Cu^2+^ in shoot till 35.0 kg P ha^–1^, while 52.5 kg P ha^–1^ decreased these nutrients compared to 35.0 kg P ha^–1^. However, P reduced Fe^3+^, Mn^2+^, and Cu^2+^ in roots and this reduction was in parallel with increasing P rate till 52.5 kg P ha^–1^. Under the interactive effect of P source and P rate, Fe^3+^, Mn^2+^, Zn^2+^, and Cu^2+^, the F test was no significant in both shoots and roots. Fe^3+^ and Mn^2+^ in shoots and its increase was gradient with increasing P rate till 52.5 kg P ha^–1^. However, the application of P in either SP or UP increased Zn^2+^ and Cu^2+^ till 35.0 kg P ha^–1^, while 52.5 kg P ha^–1^ decreased both Zn^2+^ and Cu^2+^ compared to 35.0 kg P ha^–1^. Generally, P application increased micro-nutrients in roots of common bean and this reduction was in parallel with increasing P rate.

Interactive effect of different sources and rates of P fertilization on K^+^:Na^+^ and Ca^2+^:Na^+^ ratios in shoots and roots of common bean plants grown under salt-affected soil were shown in Table 6. The significance of the F test of analysis of variance was shown in Table 6. The main effects of P source were significant (*P* ≤ 0.05) in K^+^:Na^+^ and Ca^2+^:Na^+^ ratios in shoots and UP increased significantly these ratios in both shoots and roots compared to SP. The main effects of P rates were highly significant (*P* ≤ 0.01) and affected in all aforesaid traits as shown in Table 6. In general, the application of P increased K^+^:Na^+^ and Ca^2+^:Na^+^ ratios in both shoots and roots of common bean, and this increase coincided with increasing P rate to 52.5 kg P ha^–1^. However, Ca^2+^:Na^+^ ratios in roots scored the highest value at 35.0 kg P ha^–1^. Under the interactive effect of P source and P rate, only K^+^:Na^+^ ratio in roots, the F test was highly significant (*P* ≤ 0.05). In general, UP was significantly higher or without significant difference with SP in both K^+^:Na^+^ and Ca^2+^:Na^+^ ratios either in shoots or roots.

### Growth and biomass yield of common bean

The growth parameters which included plant height, leaf area per plant, shoot dry weight (g), root dry weight (g), total dry weight per plant (g) and total dry weight per ha (t) were shown in Table 7. In addition, the significance of the F test of analysis of variance was shown in Table 7. The main effects of P source and rate were significant (*P* ≤ 0.05) on all mentioned traits. UP increased all traits compared to SP. The main effects of P rates were highly significant (*P* ≤ 0.01) and affected all aforesaid traits in Table 7. UP source of P increased total dry weight yield (t ha^–1^) by 18.7% compared to SP. Increasing P rates resulted in increasing all mentioned traits gradually to the dose of 35.0 kg P ha^–1^. For example, 35.0 kg P ha^–1^, increased total dry weight yield (t ha^–1^) by 93.8% compared to the control. Also, the interaction effect of P source and P rate was highly significant (*P* ≤ 0.05) for all mentioned traits except plant height. The interactive effects increased all growth traits, consequently increased the main target of total dry weight (t ha^–1^). All traits increased gradually with increasing P rate and application 35.0 kg P ha^–1^ either in SP or UP source increased significantly all traits. Application of 35.0 kg P ha^–1^ either in SP or UP increased total dry weight (t ha^–1^) by 84.9% and 101.9%, respectively compared to control plants. We found a significant association between leaf area per plant and total dry weight (t ha^–1^), where the correlation co-efficient was highly significant (*r* = 0.994).

### Correlation matrix

Pearson’s correlation coefficients (above diagonal) between plant dry weight (shoot dry weight and/or total dry weight per plant) and all nutrients measured in shoots of common bean plants grown with different sources and rates of phosphorus under salt-affected soil were shown in Table 8. There was a positive significant association between shoot dry weight and total dry weight with *r* = 1 (*P* ≤ 0.01). There was a positive significant correlation between total dry weight and N, P, Ca^2+^, Mg^2+^, Fe^3+^, Mn^2+^, Zn^2+^, and Cu^2+^, which were associated with one another. Besides, there was a negative association between either shoot dry weight or total dry weight and Na^+^ (*P* ≤ 0.01).

**Table 8:** Pearson’s correlation coefficients (above diagonal) among plant dry weight (shoot dry weight and total dry weight per plant) and all measured nutrients in the shoot of common bean plants grown under salinity stress (*n* = 8).

Trait	N	P	K^+^	Ca^2+^	Mg^2+^	Na^+^	Fe^3+^	Mn^2+^	Zn^2+^	Cu^2+^	SDW	TDW
N	1	0.844**	0.493^ns^	0.640^ns^	0.847**	−0.770*	0.835**	0.752*	0.893**	0.852**	0.813**	0.819**
P		1	0.223^ns^	0.859**	0.710*	−0.900**	0.833**	0.912**	0.917**	0.836**	0.980**	0.980**
K^+^			1	-0.240^ns^	0.816**	0.129^ns^	−0.045^ns^	−0.108^ns^	0.412^ns^	0.607^ns^	0.198^ns^	0.211^ns^
Ca^2+^				1	0.296^ns^	−0.969**	0.903**	0.942**	0.659^ns^	0.458^ns^	0.819**	0.813**
Mg^2+^					1	−0.428^ns^	0.475^ns^	0.432^ns^	0.803*	0.923**	0.678^ns^	0.688*
Na^+^						1	−0.962**	−0.981**	−0.775*	−0.597^ns^	−0.878**	−0.874**
Fe^3+^							1	0.904**	0.722*	0.565^ns^	0.785*	0.784*
Mn^2+^								1	0.835**	0.649^ns^	0.923**	0.918**
Zn^2+^									1	0.944**	0.953**	0.956**
Cu^2+^										1	0.857**	0.864**
SDW											1	1.000
TDW												1

**Note:**

**and *, significant at 0.01 and 0.05 levels, respectively; ns, non-significant. N, nitrogen; P, phosphorus; K^+^, potassium; Ca^2+^, calcium; Mg^2+^, magnesium; Na^+^, sodium; Fe^3+^, iron; Mn^2+^, manganese; Zn^2+^, zinc; Cu^2+^, copper; SDW, shoot dry weight; TDW, total dry weight per plant.

Table 9 showed Pearson’s correlation coefficients (below diagonal) between plant dry weight (root dry weight and total dry weight) and all nutrients measured in roots of common bean plants fertilized with different sources and rates of phosphorus and grown under salt-affected soil. There was a positive significant association between root dry weight and total dry weight with *r* = 1 (*P* ≤ 0.01). There was a positive significant correlation between total dry weight and each of P, K^+^, Ca^2+^, and Zn^2+^, which were associated with one another. Also, there was a negative association between total dry weight and each of N, Na^+^, Fe^3+^, Mn^2+^, and Cu^2+^ (*P* ≤ 0.01).

**Table 9:** Pearson’s correlation coefficients (below diagonal) among plant dry weight (root dry weight and total dry weight per plant) and all measured nutrients in the root of common bean plants grown under salinity stress (*n* = 8).

Trait	N	P	K^+^	Ca^2+^	Mg^2+^	Na^+^	Fe^3+^	Mn^2+^	Zn^2+^	Cu^2+^	RDW
N	1										
P	−0.973**	1									
K^+^	−0.483^ns^	0.309^ns^	1								
Ca^2+^	−0.528^ns^	0.451^ns^	0.855**	1							
Mg^2+^	−−0.789*	0.717*	0.508^ns^	0.346^ns^	1						
Na^+^	0.990**	−0.981**	−0.395^ns^	−0.459^ns^	−0.777*	1					
Fe^3+^	0.981**	−0.974**	−0.488^ns^	−0.592^ns^	−0.790*	0.965**	1				
Mn^2+^	0.977**	−0.971**	−0.502^ns^	−0.615^ns^	−0.776*	0.960**	0.999**	1			
Zn^2+^	−0.935**	0.860**	0.607^ns^	0.566^ns^	0.823**	−0.912**	−0.895**	−0.890**	1		
Cu^2+^	0.984**	−0.944**	−0.478^ns^	−0.495^ns^	−0.777*	0.974**	0.944**	0.938**	−0.972**	1	
RDW	−0.801*	0.753*	0.714*	0.855**	0.514^ns^	−0.746*	−0.818**	−0.833**	0.828**	−0.804*	1
TDW	−0.871**	0.839**	0.661^ns^	0.809*	0.562^ns^	−0.823**	−0.891**	−0.904**	0.840**	−0.853**	0.983**

**Note:**

**and *, significant at 0.01 and 0.05 levels respectively; ns, non-significant. N, nitrogen; P, phosphorus; K^+^, potassium; Ca^2+^, calcium; Mg^2+^, magnesium; Na^+^, sodium; Fe^3+^, iron; Mn^2+^, manganese; Zn^2+^, zinc; Cu^2+^, copper; RDW, root dry weight; TDW, total dry weight.

### Response curve of biomass yield to phosphorus fertilizer rate

**Figure 1:**
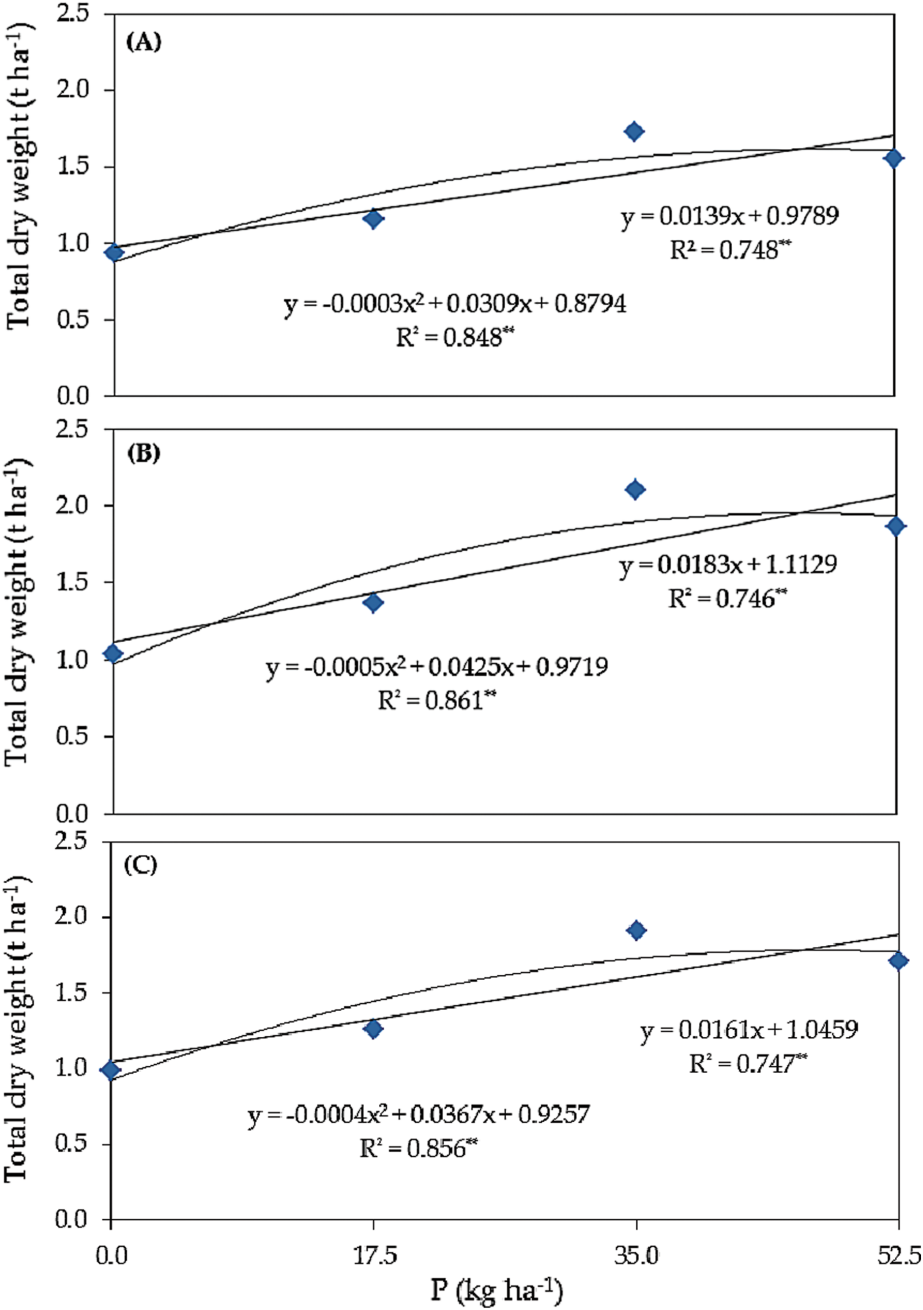
Response curve of total dry weight (t ha^−1^) to different rates of phosphorus (kg ha^−1^) using different forms of phosphorus i.e. super superphosphate (A), urea phosphate (B), and at an average combined of superphosphate and urea phospha (C) in common bean plants grown under salinity stress.

Linear and quadratic responses of common bean plants total dry weight (t ha^–1^) grown under salinity stress to different rates of phosphorus (kg ha^–1^) using different forms of phosphorus, i.e., superphosphate (A), urea phosphate (B), and at an average combined of superphosphate and urea phosphate (C) are shown in Fig. 1. Under superphosphate (A), with a P level increased of 1.0 kg ha^–1^, the total dry weight was expected to increase by 14 kg ha^–1^. The R^2^ value, defined as the regression sum of squares divided by the total sum of squares, increased from 74.8% (linear) to 84.8% (quadratic), so 84.8% of the variation in total dry weight yields was explained by quadratic regression model. In the quadratic curve, total dry weight, equal 1.675 t ha^–1^, was the maximum if P was applied at a level of 51.5 kg ha^–1^, and the expected TDW should be 1.675 t ha^–1^. Under urea phosphate (B), with a P level increase of 1.0 kg ha^–1^, the total dry weight was expected to increase by 18 kg ha^–1^. The mentioned value increased from 74.6% (linear) to 86.1% (quadratic). Thus, if P was applied at a level of 42.5 kg ha^–1^, the maximum expected total dry weight would be 1.875 t ha^–1^. Under average combined of superphosphate and urea phosphate (C), with a P level increased of 1.0 kg ha^–1^, the total dry weight was expected to increase by 16 kg ha^–1^. This has increased from 74.7% (linear) to 85.6% (quadratic). Hence, if P was applied at a level of 45.9 kg ha^–1^, the maximum expected total dry weight would be 1.768 t ha^–1^.

## Discussion

Leaf chlorophyll is one of the most important physiological measures of biochemical characteristics, indicating the stress of the plant, partly because of its dependence on water and nutritious accessibility (Bargaz et al., 2016; El-Lethy, Abdelhamid & Reda, 2013). The photosynthetic pigments content decrease in conditions of salinity stress (Bargaz et al., 2016). In the present study, the reduced total chlorophyll (TC) content was confirmed as a result of salinity stress affecting common bean plants metabolism. Likewise, the carotenoids (Car) concentration was reduced in parallel (Table 2). Additionally, there was a significant linear correlation between the total chlorophyll content in the leaves and total dry weight yield per hectare (*r* = 0.777, *P* ≤ 0.01), as well as between total carotenoid and total dry weight yield per hectare (*r* = 0.778, *P* ≤ 0.01). The decrease in chlorophylls’ content in plants grown in stress conditions could be attributable to thylakoid membrane disorganization, and to degradation of chlorophyll caused by proteolytic enzymes such as chlorophyllase (Rong-Hua et al., 2006). Mentioned processes could lead to a decrease of the net assimilation rate and—in consequence relative growth rate of plants (Rady et al., 2015). The inhibitory effect of accumulated Na^+^ ions could also participate in described phenomenon (Abdelhamid, Shokr & Bekheta, 2010; Dawood, Abdelhamid & Schmidhalter, 2014). The improvement in the content of chlorophyll and carotenoids due to P application may be due to more effective absorption of elements from soil, particularly Mg, a key component of the chlorophyll molecule (Sheng et al., 2008). In short, a higher content of chlorophyll in plants contributes to accelerated photosynthetic activity and more intensive growth and higher dry weight yield.

In stressed plants, the reduction in nucleic acids (DNA and RNA) can be attributed to the reactive oxygen species (ROS) released to cell components in salt stress conditions. ROS induce DNAase activity, accelerating DNA fragmentation (Sadak, Abdelhamid & Schmidhalter, 2015; Tsenov, Strogonov & Kabanov, 1973). The content of DNA and RNA in maize seedlings has been postulated to be decreased under salt stress due to inhibition of nucleic acids synthesis and intensification of their breakdown (Zeid, 2009b). Plant growth, directly associated with cell division, diversification, and elongation were negatively affected by salinity through reduction of the cellular water content and decreasing of nucleic acids, which are crucial for all anabolic processes. In bean, Zeid (2009a) determined that the decrease in nucleic acids content was linked to accelerated RNase activity causing their degradation. Kabanov & Aziyashvili (1976) associated the depression of DNA and RNA metabolism to the abundance of Na^+^ cations in cells, reduction of phosphorus incorporation into nucleic acids and also to intensified RNase activity in cytoplasm. In the present study, a significant linear correlation was determined between the RNA in the leaves of common bean and total dry weight yield per hectare (*r* = 0.985, *P* ≤ 0.01), and between DNA and total dry weight yield per hectare (*r* = 0.954; *P* ≤ 0.01). Moreover, the application of P using both P sources promoted the synthesis of nucleic acids and could prevent their degradation by nuclease enzymes (Table 3).

Plant growth is based on a sufficient supply of N transformed to form amino acids, proteins, nucleic acids, and other cellular components for various parts of the plant and translocated into the plant kernels that develop intensively. P and K^+^ are involved in mitigating the salinity stress in most of plant crops (El-Lethy, Abdelhamid & Reda, 2013; Bargaz et al., 2016; Chakraborty et al., 2016). For example, P, K^+^, and indole-3-acetic acid (IAA) were effective in improving the maize plants fitness while exposed to the salinity stress (Kaya et al., 2013). Besides, it was reported that P application at a doses of 50, 100, and 150 mg g^−1^ increased root length, cotyledon length and width, and hypocotyls length, contents of N, P, Mn^2+^, and Cu^2+^ in shoot and P, Ca^2+^ in the root, and decreased Fe^3+^ and Zn^2+^ in the shoot, Fe^3+^, Mn^2+^, Zn^2+^, and Cu^2+^ in the root of pepper plants (Çimrin et al., 2010). Also, a significant reduction in N, P, K^+^, Ca^2+^, and Mg^2+^, while Na^+^ and Cl^‒^ increased were found in salinity stress conditions in faba bean plants (Abdelhamid, Shokr & Bekheta, 2010; Rady et al., 2015). The decrease of N, P, and K^+^was observed in common bean plants irrigated with different concentrations of NaCl, while Na^+^ increased in tissues (Dawood, Abdelhamid & Schmidhalter, 2014; Sofy et al., 2020b). In the present study, the highest rate of P (52.5 kg ha^−1^) resulted in a significant reduction of Na^+^ content in the shoot and root of common bean. These findings are similar to those reporting that various levels of P applied as a foliar spray caused a reduction in the content of Na^+^ in the shoot of wheat plants (Ghonaim, Mohamed & Omran, 2021). In sandy and calcareous soils, application of P alleviated the stressful effect of moderate and high salinity in wheat plants, while N, P, K^+^, and Zn^2+^ absorption increased (Wagdi et al., 2013).

High cellular K^+^:Na^+^ ratio, crucial for balanced cell processes, can be maintained in salinity stress conditions also through enhanced uptake of K^+^ and/or Ca^2+^ causing reduced uptake of Na^+^ (Ghonaim, Mohamed & Omran, 2021; Kavitha et al., 2012).

The present study revealed that in the shoot of common bean, the highest value in N, P, K^+^, Mg^2+^, Zn^2+^, and Cu^2+^ were obtained using 35.0 kg P ha^–1^ compared to the other rates of P fertilizer. Also, the 35.0 kg P ha^−1^ gave the highest value on N, K^+^, and Ca^2+^ in the root of the common bean. The highest P application rate of 52.5 kg ha^−1^ resulted in a significant reduction in Na^+^ in shoot and root. These findings are similar to the report that N, P, K^+^, Zn^2+^, Fe^3+^, and Mn^2+^ increased in two common bean recombinant inbred lines (RILs) 115 (P-deficiency tolerant) and 147 (P-deficiency susceptible) with higher P concentrations (Kandil, Gad & Abdelhamid, 2013). Also, the application of P caused higher N absorption and accumulation in chickpea plants (Shukla, Singh & Deshbhratar, 2010).

The dose and salt dependent P action as plant stress-defender was confirmed in some research. P fertilizer at a dose of 40 kg ha^–1^ increased N, P, K^+^, and Ca^2+^ content in maize (Hussaini et al., 2008). P at a rate of 60 kg ha^–1^ of triple superphosphate enhanced N, P, Ca^2+^, and K^+^ accumulation in lentil (Al-Darkazli, Al-Saedy & Al-Saadi, 2011). Similar results were reported by researchers for many crops, like barley (Mehrvarz & Chaichi, 2008) and faba bean (El-Gizawy & Mehasen, 2009). Moreover, P supported the development of a strong root system of lentil plants causing better capacity to absorb N, P, K^+^, and Ca^2+^ from the soil, and, consequently contents of mentioned minerals increased after the phosphorus application (Singh & Singh, 2016). High P fertilization promoted vegetative growth and the formation of well-developed root systems that efficiently absorb maximum soil nutrients (Sharma & Gupta, 2002).

Çimrin et al. (2010) pointed out that humic acid and P application positively affected the growth of pepper seedlings. Moreover, humic acid and P interacted in increasing N, P, K^+^, Ca^2+^, Mg^2+^, S, Mn^2+^, and Cu^2+^ contents of pepper seedlings’ shoot. The application of chemical P enhanced plant growth parameters and raised N, P, and K^+^ content in eggplant compared to the organic P fertilizer (Lima et al., 2014). Besides, P in any form increased the nutritional values of common bean, i.e., protein, N, P, K^+^, Mn^2+^, Zn^2+^, Cu^2+^, and Fe^3+^ contents (Bargaz et al., 2016).

Phosphorus is crucial for cell division, resulting in an increased growth, number of side branches, and in consequence, the dry weight of the plant (Tesfaye et al., 2007). Hasan et al. (2013) noted that the maximum growth parameters of brinjal were at 60 kg P ha^–1^. Whilst, Attar et al. (2012) found that 90 kg ha^−1^ phosphorus pentoxide applied to the sandy soils, resulted in optimum growth parameters of the common bean, while control plants showed the lowest growth values. Also, it was reported that the application of P improved both, growth parameters and yield, of mung bean (Abdalsalam & Al-Shebani, 2010). The plant height and dry weights of shoot and roots decreased in plants grown in high salinity conditions which is in line with previous reports (Awad et al., 2012; El-Beltagi et al., 2013; El-Lethy, Abdelhamid & Reda, 2013; Naeem et al., 2020). Our findings are also in agreement with those of Khan et al. (2013). The highest value of shoot dry weight of plants treated with P was possibly due to the significant increased the area of the leaf per plant, which increased the area available for photosynthesis and biomass production.

In this study, the decline in total dry weight yield can be due to decreased photosynthetic pigments and nucleic acids content (Tables 2 and 3), disruption in nutrient balance (Tables 4 and 5) and reduced plant growth (Table 7). In this research, in a linear way, LA and total dry weight yield per ha were correlated with each other. P application at 35.0 kg ha^−1^ rate caused significant increases in total dry weight yield compared to the corresponding control. Our finding confirmed that high rate of P (52.5 kg P ha^−1^) had harmful effects on common bean plants. In this regard, a detrimental reverse effect of high P rate was recorded in common bean (Bargaz et al., 2016) and other crops such as *Oryza sativa* (Aslam et al., 1996), which does not make the available salinity and P interactive effect data adequately consistent and a large number of grain and forage legumes need to be considered at large P levels.

## Conclusions

In conclusion, the results obtained from this study have demonstrated that phosphorus fertilization significantly increased all growth parameters, chlorophyll content, nucleic acid content and minerals content of the common bean plants. Moreover, phosphorus applied at a dose of 35.0 kg P ha^−1^ was more efficient compared to other treatments. Mentioned P dose resulted in the highest values of investigated parameters crucial for common bean growth and productivity, including chlorophylls, carotenoids, DNA, RNA, N, P, K^+^, and Mg^2+^, Zn, Cu, and K^+^:Na^+^ of shoot, N, K, Ca^2+^, K^+^:Na^+^, and Ca^2+^:Na^+^ of root, leaf area, shoot dry weight, root dry weight, total dry weight per plant, and total dry weight per hectare. The application of phosphorus as urea phosphate was more effective than single superphosphate in alleviating salinity stress.

## Supplemental Information

10.7717/peerj.11463/supp-1 Supplemental Information 1 Salinity Raw Data.

## Additional Information and Declarations

### Data Availability

The following information was supplied regarding data availability: The raw measurements are available in the Supplemental File.
